# Anatomical distribution pattern of coronary artery lesions in Kawasaki disease and the prognostic value of dynamic Z-scores for recovery time: a retrospective cohort study

**DOI:** 10.3389/fped.2026.1914785

**Published:** 2026-10-28

**Authors:** Yuhui Zhang, Yaowen Sun, Peipei Gu, Long Chen, Hui Wang, Cui Hou, Jiangnan Xu, Jundi Luo, Xuan Li, Yunjia Tang, Ling Sun, Haitao Lv, Qiu-qin Xu

**Affiliations:** 1 Department of Cardiology, Children's Hospital of Soochow University, Suzhou, Jiangsu, China; 2 Department of Urology, Yancheng First People's Hospital, Yancheng, Jiangsu, China

**Keywords:** anatomical distribution, children, coronary artery lesions, echocardiography, Kawasaki disease, prognosis, recovery time, Z-score

## Abstract

**Objective:**

To explore the anatomical distribution of coronary artery lesions (CALs) in Kawasaki disease (KD) and assess the predictive value of Z-scores at different time windows for coronary recovery time.

**Methods:**

This single-center retrospective cohort included 569 KD children diagnosed in 2021, of whom 211 had coronary dilation. Echocardiography measured Z-scores of five coronary segments. Follow-up was scheduled at diagnosis, week 2, month 1, month 3, 6 months, 1 year, and beyond. Generalized estimating equations tested inter- coronary segment correlations, and Cox regression evaluated the effect of the cumulative maximum Z-score at each time window on recovery time. Model fit was compared using AIC.

**Results:**

Lesions exhibited a statistical pattern of adjacent-segment correlation along the same vessel's longitudinal axis, strongest within the LAD and RCA systems. AIC comparison revealed that Zmax-T2 (within 2 weeks after diagnosis) provided clinically useful early prognostic information, with hazard ratios numerically similar to those of later time windows (ranging from 0.50 to 0.52). For Zmax-T2, each 1-unit increase corresponded to an approximately 49.6% lower hazard of coronary normalization (HR≈0.504). Compared with single- coronary segment involvement, recovery was significantly slower beginning in the two- coronary segment category. Female sex was associated with faster recovery (HR = 1.369, *P* = 0.041), whereas age and season showed no significant effect.

**Conclusion:**

KD coronary lesions exhibit axial adjacent-segment correlation. Zmax-T2 is a practically useful early predictor of recovery, supporting early risk stratification. The number of involved coronary segments remained associated with recovery after adjustment for Zmax-T2, although the proportional hazards assumption was not fully met for this variable, and the association should be interpreted as an average effect over the entire follow-up period.

## Introduction

Kawasaki disease (KD) is an acute systemic vasculitis of unknown etiology, primarily affecting children under 5 years of age (1). Nationwide epidemiological surveys in Japan have shown that the annual incidence rate in children under 5 years is as high as approximately 359 per 100,000 (2); in developed countries, Kawasaki disease has become the leading cause of acquired heart disease, surpassing acute rheumatic fever (3). Although the widespread use of intravenous immunoglobulin (IVIG) has significantly reduced the incidence of CAA from 25% to approximately 4%–5%, some children still develop coronary artery dilation or aneurysms, making it the most common acquired heart disease in childhood (4, 5). These coronary artery lesions, especially giant aneurysms, can lead to severe adverse cardiovascular events such as myocardial infarction and sudden death, with effects that can persist into adulthood (6).

Accurately assessing the severity of coronary artery involvement is the cornerstone for risk stratification and guiding long-term management. For a long time, clinical practice has relied primarily on the absolute internal diameter criteria of the Japanese Ministry of Health, Labour and Welfare (JMHW) for diagnosis and classification (7). However, because this standard does not account for differences in patient body size, it may underestimate early or mild coronary artery dilation (8, 9). In recent years, the body surface area (BSA)-standardized coronary artery Z-score system has been widely adopted by international guidelines, including those from the American Heart Association (AHA) and the Japanese Circulation Society (JCS), for a more precise definition of coronary artery abnormalities (CAA) and aneurysm grading (5, 7, 10). The Z-score system can better distinguish the severity of lesions and shows good correlation with clinical outcomes (11, 12).

However, the application of Z-scores in clinical practice and prognostic research still faces challenges and knowledge gaps. First, different Z-score calculation formulas (e.g., Dallaire, Kobayashi, Lopez), established based on different populations and statistical methods, may yield significantly different Z-scores for the same measurement. This inconsistency, especially in larger aneurysms, can affect diagnosis and treatment decisions (13, 14). Second, current research mostly focuses on the relationship between Z-scores at a single time point (e.g., at diagnosis or peak of the disease course) and prognosis (11, 15). Coronary artery lesions in Kawasaki disease are a dynamic evolving process. The severity of lesions may differ across various time windows from the acute inflammatory phase to the subacute and chronic phases. Furthermore, no study has systematically compared the prognostic value of Z-scores at different follow-up time points (e.g., acute phase, subacute phase) for long-term recovery. Identifying a clinically useful assessment time window would greatly optimize follow-up strategies and resource allocation.

Additionally, the understanding of the spatial distribution pattern of lesions remains incomplete. Involvement of multiple coronary artery segments is a common clinical phenomenon and is associated with poor prognosis (10, 16). Clinical observations suggest that the left anterior descending artery (LAD) and right coronary artery (RCA) are more frequently involved. However, it is unclear whether this distribution is random or follows a specific anatomical adjacent-segment correlation pattern (e.g., adjacent-segment correlation along the vessel axis), as statistical evidence based on large sample data is lacking. Identifying this pattern would not only aid in understanding the disease's pathophysiology but also provide a basis for targeted imaging assessment.

To address these gaps, this retrospective cohort study aimed to: 1) use a Generalized Estimating Equation (GEE) model to assess the inter-segment correlation and anatomical distribution pattern of disease risk across coronary segments within the same patient; 2) evaluate the relative model fit of cumulative maximum Z-scores at diagnosis, within 2 weeks after diagnosis, within 1 month after diagnosis, and within 3 months after diagnosis for coronary recovery time, and identify a practically useful early assessment window; and 3) comprehensively evaluate the impact of lesion extent (number of involved coronary segments) and demographic factors on prognosis.

## Methods

### Study design and patient cohort

This single-center retrospective study consecutively enrolled all patients diagnosed with Kawasaki disease (KD) at the Children's Hospital of Soochow University between January 1 and December 31, 2021, with no exclusions based on disease severity, laboratory findings, or treatment response (including 211 patients with coronary artery dilation). The Children's Hospital of Soochow University serves as a tertiary referral center for pediatric cardiology in the Jiangsu region, which may result in overrepresentation of patients with more severe or complicated disease compared with primary care or community-based cohorts. All descriptive and intergroup comparisons were performed on the total cohort of 569 patients, while analyses of inter-segment correlation and recovery time were restricted to the 211 patients with coronary artery dilation. Diagnosis was based on the latest American Heart Association (AHA) guidelines. Coronary artery involvement was defined as a body surface area (BSA)-adjusted Z-score ≥2.0 in any segment, calculated using the Dallaire formula (17). This study was approved by the Institutional Review Board.

Follow-up protocol and outcome definition: All patients underwent systematic echocardiographic follow-up according to a standardized protocol, with assessments scheduled at the initial diagnostic evaluation (T1), which was performed immediately after KD diagnosis, typically on the same day, week 2 (T2), month 1 (T3), month 3 (T4), and subsequently at 6 months, 1 year, and beyond 2 years as clinically indicated. Follow-up intervals were individualized based on lesion severity in accordance with the American Heart Association and Japanese Circulation Society guidelines, with more frequent assessments for patients with moderate to giant aneurysms. The primary outcome was defined as normalization of coronary artery diameter, strictly requiring at least two consecutive echocardiographic examinations showing a Z-score < 2.0 in all measured coronary segments. The recovery date was defined as the date of the first echocardiogram showing Z < 2.0 in all segments, with the subsequent confirmatory examination serving to validate sustained normalization. In the two patients who experienced transient normalization followed by recurrent dilation, the recovery date was defined as the date of the final confirmed normalization. Patients who had not achieved normalization by the study cutoff date were treated as censored observations, with the censoring time defined as the date of the last available echocardiographic examination. These censored patients had a median follow-up duration of 980 days (IQR 763.75–1,051.75 days), indicating that they had completed nearly three years of follow-up on average, and they continue to undergo scheduled follow-up evaluations beyond the study cutoff date. No patients were lost to follow-up.

### Coronary artery assessment and data collection

All echocardiographic examinations were performed by two experienced pediatric cardiac ultrasound specialists using a standardized operating procedure. Examinations were performed using GE Vivid E90, Philips EPIQ 7C, or Philips iE33 ultrasound systems, each equipped with a phased-array transducer (frequency range 3–8 MHz, adjusted according to patient body size). All measurements were obtained from digitally stored images with offline calibration. Coronary artery internal diameters were measured at end-diastole (timed to the peak of the R-wave on the simultaneous electrocardiogram), using the inner edge-to-inner edge technique. For each of the five segments, the segment identity was determined by standard anatomical landmarks. In non-dilated segments, measurements were taken at the specific sites originally described by Dallaire and Dahdah: LMCA (midway between the ostium and the bifurcation), LAD, LCX, and proximal RCA (3–5 mm distal to their origin), and RCAmed (subcostal view at the lateral aspect of the atrioventricular sulcus). In dilated or aneurysmal segments, the maximal internal diameter within the affected segment was measured to capture the peak lesion burden, while carefully avoiding the ostium and bifurcation sites unless the lesion itself involved these structures. Figure 1 illustrates the measurement sites for each coronary segment using a schematic diagram and corresponding echocardiographic images. The five segments were selected because they represent the standard coronary segments recommended by the American Heart Association for echocardiographic assessment in Kawasaki disease, and because they are the segments for which the Dallaire Z-score formula was originally validated, including the LCX and RCAmed in the original derivation cohort (17). All measurements were performed by two experienced echocardiographers following a strictly standardized protocol, with blinding to clinical data. Formal inter- and intra-observer reproducibility analyses were not pre-specified in this retrospective study; however, the use of a standardized protocol and experienced operators minimizes measurement bias. The follow-up protocol included systematic examinations at diagnosis (T1), week 2 (T2), month 1 (T3), and month 3 (T4) of the disease course, with increased follow-up frequency based on clinical needs. For each examination, the internal diameters and calculated Z-scores of the following five coronary artery segments were precisely measured and recorded: left main coronary artery (LMCA), left anterior descending artery (LAD), left circumflex artery (LCX), right coronary artery main (RCA), and right coronary artery mid-segment (RCAmed).

**Figure 1:**
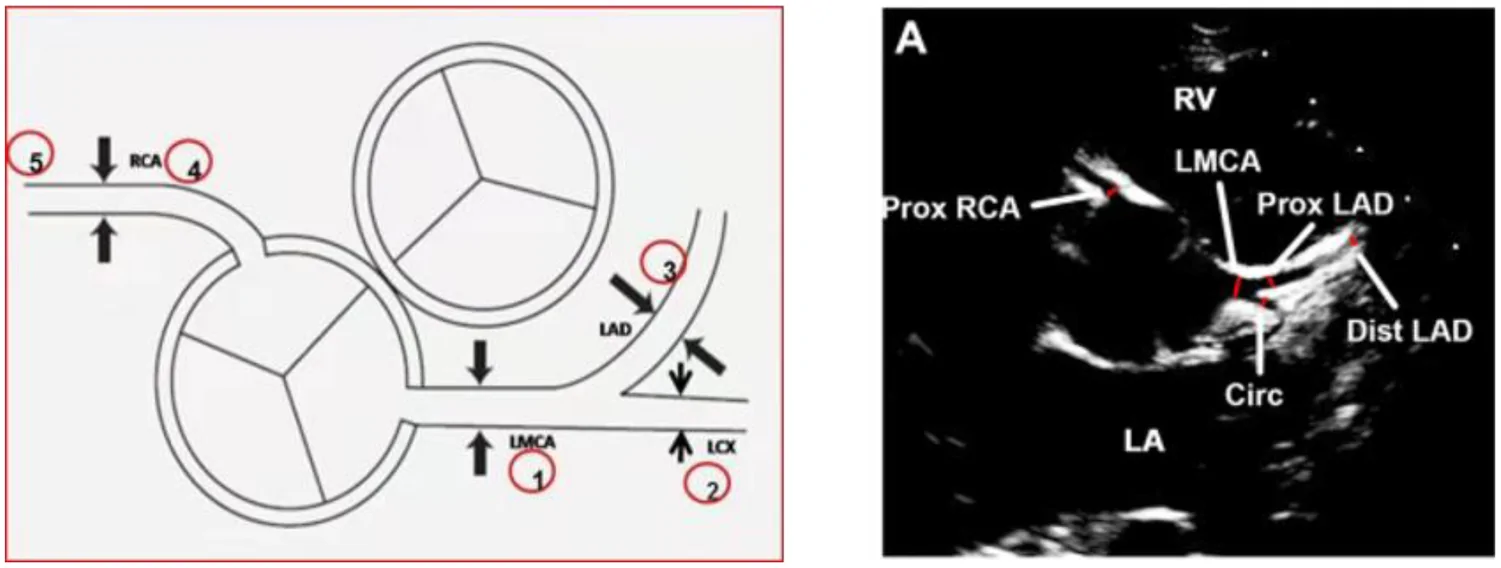
Representative schematic and echocardiographic images showing the measurement sites of coronary artery segments in Kawasaki disease. Left panel: schematic diagram illustrating the standard coronary segments assessed in this study. Right panel: corresponding echocardiographic images demonstrating the measurement sites. LMCA, left main coronary artery; LAD, left anterior descending artery; LCX, left circumflex artery; RCA, right coronary artery; RCAmed, mid-segment of the right coronary artery; Ao, aorta; LA, left atrium; LV, left ventricle; RV, right ventricle; RA, right atrium; RPA, right pulmonary artery. Yellow arrowheads indicate the inner-edge-to-inner-edge measurement sites at end-diastole.

Key variables were defined as follows: For each echocardiographic examination, the maximum Z-score among the five coronary segments was recorded as the visit-specific maximum Z-score. For each time window, the cumulative maximum Z-score was defined as the highest value among all follow-up records from diagnosis up to that time point: T1 (at diagnosis), T2 (within 2 weeks after diagnosis), T3 (within 1 month after diagnosis), and T4 (within 3 months after diagnosis), designated as Zmax-T1 through Zmax-T4, respectively. The overall absolute maximum value recorded during the entire follow-up period was defined as the global Zmax (Zmax-Global). This cumulative definition ensures that Zmax-T2, T3, and T4 represent the peak lesion severity accumulated by each time window rather than a single time-point measurement. Visit-specific maximum Z-scores were used to describe the dynamic trajectory of lesions and as predictors in the landmark analyses, whereas cumulative maximum Z-scores were used in all primary time-window Cox proportional hazards models.

The number of involved coronary segments was defined as the maximum number of segments with a Z-score ≥2.0 observed at any time during the entire follow-up period. Because proximal and mid-RCA segments were counted separately, this variable represents the number of involved coronary segments rather than the number of distinct coronary arteries.

The primary outcome of the study was the “normalization of coronary artery internal diameter,” strictly defined as: all measured coronary artery segments in the same patient having a persistent Z-score < 2.0. Survival time was defined as the number of days from the date of disease diagnosis to the date of coronary artery normalization. For patients who had not recovered by the study end date, their data were treated as censored data, with the censoring time being the date of the last follow-up.

### Treatment protocol

All patients received intravenous immunoglobulin (IVIG) at a dose of 2 g/kg as first-line therapy, administered within 10 days of illness onset, in accordance with the American Heart Association guidelines and the Chinese consensus on Kawasaki disease management. Patients who failed to defervesce within 36–48 h after completion of initial IVIG were considered IVIG-resistant and received additional anti-inflammatory therapy at the discretion of the attending physician. Detailed data on IVIG resistance and adjunctive therapies were not systematically captured for this retrospective cohort, as this was not a prespecified aim of the study.

### Statistical analysis

This study used IBM SPSS Statistics (version 26.0) for statistical analysis. R language (version 4.3.2) was used to assist with survival analysis using the single coronary segment as the unit of analysis.

#### Data preprocessing and missing value imputation

Among the 211 patients with coronary artery dilation, the proportion of missing visit-specific maximum Z-scores increased progressively during follow-up: 0/211 (0%) at diagnosis (T1), 2/211 (0.9%) at week 2 (T2), 8/211 (3.8%) at month 1 (T3), and 24/211 (11.4%) at month 3 (T4). To handle these missing data while preserving statistical power and minimizing bias, multiple imputation was performed on the visit-specific maximum Z-scores using the built-in Multiple Imputation module in SPSS, with predictive mean matching (PMM) as the imputation method. The imputation model included all variables used in subsequent analyses: age, sex, visit-specific maximum Z-scores at T1–T4, number of involved coronary segments, follow-up time, and the censoring indicator. Five imputed datasets were generated with 10 iterations, and convergence was assessed by visual inspection of trace plots. After imputation, cumulative maximum Z-scores (Zmax-T1 to Zmax-T4) were calculated from the imputed visit-specific maximum Z-scores for use in the Cox proportional hazards models. For the segment-level analysis, two-level multiple imputation was performed in R to preserve the clustering structure of repeated measurements within the same patient, using the same set of predictors. To assess the robustness of our findings, we performed a complete-case sensitivity analysis using only patients with complete visit-specific Z-score data across all time windows, from which cumulative maximum Z-scores (Zmax-T1 to Zmax-T4) were calculated. The results were consistent with those obtained from the multiple imputation approach (all HRs ranging from 0.52 to 0.62, *P* < 0.001), supporting the robustness of our conclusions. Multiple imputation was therefore used as the primary analytical approach, consistent with current recommendations for handling missing data in observational studies.

#### Descriptive statistics and intergroup comparisons

All descriptions and intergroup comparisons were performed in SPSS. Continuous variables (e.g., age, Z-score) were presented as median (interquartile range, IQR). Intergroup comparisons were performed using the Kruskal–Wallis H test. If the grouping variable was ordinal (e.g., lesion severity grade), the Jonckheere–Terpstra test was further used to analyze trends (*P*-trend reported). Categorical variables (e.g., sex, season, segment involvement) were presented as frequency (percentage). Intergroup comparisons were performed using Pearson's chi-square test; when more than 20% of cells had an expected count less than 5, Fisher's exact test or the Fisher-Freeman-Halton exact test was used instead. For ordinal grouping variables, the linear-by-linear association test was used to analyze trends (*P*-trend reported).

#### Inter-segment correlation analysis

A Generalized Estimating Equation (GEE) model was used to analyze the risk of coronary segment involvement, with an unstructured working correlation matrix to adjust for intra-individual correlation.

#### Survival analysis (analysis of factors influencing recovery time)

With the patient as the unit of analysis: Univariate Cox proportional hazards regression models (Enter method) were constructed for each time window (Zmax-T1 through T4 and Zmax-Global) to evaluate the predictive effect of each Zmax variable on recovery time. To compare the model fit across time windows, the Akaike Information Criterion (AIC) was calculated for each univariate model using the formula AIC = −2 log likelihood + 2k, where k = 1 for each univariate model. A smaller AIC value indicates better model fit, and differences in AIC (*Δ*AIC) were interpreted as follows: *Δ*AIC < 2 indicates no meaningful difference; 2–6 indicates positive evidence; 6–10 indicates strong evidence; and >10 indicates very strong evidence in favor of the model with the smaller AIC. Additionally, to evaluate whether the number of involved coronary segments remained associated with recovery after adjustment for Zmax-T2, a multivariable Cox model was constructed by simultaneously entering Zmax-T2 and coronary segment count as covariates (Enter method). Coronary segment count was entered as a continuous variable (number of involved coronary segments, ranging from 1 to 5) to estimate the effect per one-segment increase. Multicollinearity was assessed using the variance inflation factor (VIF), with VIF < 5 indicating no significant collinearity. The proportional hazards (PH) assumption was evaluated using Schoenfeld residuals. When the test suggested evidence of time-varying effects (*P* < 0.05), we extended the standard Cox model to allow for time-varying coefficients by incorporating a log(time) interaction term, using the Cox regression with time-dependent covariate procedure in SPSS (equivalent to the tt() function in R). This time-varying coefficient analysis was performed for Zmax-T2 only, because coronary segment count is a count variable (1–5 coronary segments) for which modeling a time-varying coefficient poses substantial challenges in both estimation and interpretation. This approach models the hazard ratio as a function of follow-up time, providing a more accurate description of the dynamic prognostic effect. The standard Cox model was retained to estimate the overall (average) effect over the entire follow-up period, while the time-varying coefficient model was used to characterize the dynamic evolution of the effect over time.

With the individual coronary segment as the unit of analysis: To handle the clustered data of recovery times for multiple coronary segments within the same patient, a Cox proportional hazards model with a gamma-distributed frailty term was constructed using the survival package in R language to incorporate individual-level random effects. In all Cox proportional hazards models, the event was coded as coronary artery normalization (recovery), and the reference group for sex was set as male.

#### Landmark analysis

To address the potential time-dependent bias inherent in using later-window Zmax variables (T3 and T4) to predict recovery time counted from diagnosis, we performed landmark analyses at four prespecified time points: diagnosis (T1, day 0), 2 weeks (T2, day 14), 1 month (T3, day 30), and 3 months (T4, day 90). At each landmark time point, we included only patients who had not yet achieved normalization and remained under observation at that time point. Patients who recovered before the landmark time point were excluded. The survival time was recalculated from the landmark time point to the date of subsequent recovery (i.e., landmark_survival_time = recovery_time−landmark_day), and censoring was defined as no recovery after the landmark time point. The visit-specific maximum Z-score at each landmark visit (diagnosis, 2 weeks, 1 month, and 3 months, respectively) was used as the predictor in separate Cox proportional hazards models. This approach ensures that the predictive effect of Zmax is evaluated only among patients who remained at risk at each time point, thereby eliminating the immortal time bias that would arise from including patients who had already recovered before the measurement window.

#### Reproducibility analysis

To assess inter-observer reproducibility, two experienced echocardiographers independently measured coronary artery diameters in a randomly selected subset of 40 patients (approximately 19% of the dilated cohort) using archived echocardiographic images from the initial diagnostic evaluation (T1). Intraclass correlation coefficients (ICC) were calculated using a two-way mixed effects model with absolute agreement, based on single measurements. An ICC >0.75 was considered indicative of good reproducibility, and >0.90 as excellent.

#### Statistical significance

All hypothesis tests were two-sided, and a *P*-value <0.05 was considered statistically significant.

## Results

### Patient baseline characteristics and lesion severity grouping

A total of 569 patients diagnosed with Kawasaki disease were included in this study. According to the American Heart Association (AHA) classification criteria for the severity of coronary artery lesions, the patient baseline characteristics and lesion severity are detailed in Table 1. The patient flow diagram is presented in Figure 2.

**Table 1:** Baseline characteristics and coronary artery lesion dynamics by severity groups (*N* = 569 patients diagnosed with Kawasaki disease).

Variable	Normal (zMAX <2) (*n* = 358)	Dilation (2 ≤ zMAX < 2.5) (*n* = 71)	Small Aneurysm (2.5 ≤ zMAX < 5) (*n* = 119)	Medium Aneurysm (5 ≤ zMAX < 10) (*n* = 13)	Giant Aneurysm (zMAX ≥10) (*n* = 8)	*P*-value (Overall)	*P*-trend
Demographics							
Age (months), median [IQR]	32.50 (18.00–56.00)	25.00 (16.00–47.50)	20.00 (12.00–45.00)	26.00 (10.00–69.00)	47.00 (21.50–50.00)	0.002	0.007
Male sex, *n* (%)	214 (59.8%)	49 (69.0%)	80 (67.2%)	12 (92.3%)	6 (75.0%)	0.058	–
Season of Onset, *n* (%)							
Spring (Mar-May)	94 (26.3%)	16 (22.5%)	32 (26.9%)	2 (15.4%)	4 (50.0%)	0.356	–
Summer (Jun-Aug)	81 (22.6%)	17 (23.9%)	37 (31.1%)	3 (23.1%)	0 (0.0%)		
Autumn (Sep-Nov)	102 (28.5%)	26 (36.6%)	33 (27.7%)	5 (38.5%)	2 (25.0%)		
Winter (Dec-Feb)	81 (22.6%)	12 (16.9%)	17 (14.3%)	3 (23.1%)	2 (25.0%)		
Coronary Artery Z-Score Dynamics							
Initial zMAX, median [IQR]	1.22 (0.80–1.54)	2.19 (2.03–2.31)	2.82 (2.55–3.42)	5.20 (4.30–6.11)	9.93 (3.63–12.15)	<0.001	<0.001
zMAX at 2-week follow-up, median [IQR]	1.11 (0.75–1.47)	2.03 (1.68–2.24)	2.65 (2.32–3.24)	5.19 (4.73–6.53)	10.90 (10.31–14.89)	<0.001	<0.001
zMAX at 1-month follow-up, median [IQR]	0.91 (0.58–1.26)	1.67 (1.34–1.88)	2.21 (1.72–2.75)	5.67 (4.29–6.83)	12.89 (10.46–17.12)	<0.001	<0.001
zMAX at 3-month follow-up, median [IQR]	-	1.25 (0.91–1.60)	1.67 (1.16–2.02)	3.87 (2.39–6.42)	11.64 (10.19–16.93)	<0.001	<0.001
Peak zMAX (entire course), median [IQR]	-	2.23 (2.13–2.35)	2.97 (2.69–3.63)	6.10 (5.67–7.15)	12.89 (11.02–18.11)	<0.001	<0.001
Coronary Segments involved, *n* (%)							
Left main (LMCA)	-	19 (26.8%)	55 (46.2%)	9 (69.2%)	4 (50.0%)	0.006	0.003
Right coronary (RCA)	-	17 (23.9%)	54 (45.4%)	10 (76.9%)	5 (62.5%)	<0.001	<0.001
Left anterior descending (LAD)	-	40 (56.3%)	91 (76.5%)	13 (100.0%)	8 (100.0%)	<0.001	<0.001
Left circumflex (LCX)	-	2 (2.8%)	8 (6.7%)	5 (38.5%)	5 (62.5%)	<0.001	<0.001
Middle segment of the right coronary artery(RCAmed)	-	18 (25.4%)	57 (47.9%)	10 (76.9%)	8 (100.0%)	<0.001	<0.001
Multi-segment disease (≥2 segments), *n* (%)	-	20 (28.2%)	82 (68.9%)	12 (92.3%)	8 (100.0%)	<0.001	<0.001
1 coronary segment	-	51 (71.8%)	37 (31.1%)	1 (7.7%)	0 (0.0%)	<0.001	<0.001
2 coronary segments	-	16 (22.5%)	35 (29.4%)	1 (7.7%)	0 (0.0%)		
3 coronary segments	-	3 (4.2%)	32 (26.9%)	4 (30.8%)	4 (50.0%)		
4 coronary segments	-	1 (1.4%)	13 (10.9%)	3 (23.1%)	2 (25.0%)		
5 coronary segments	-	0 (0.0%)	2 (1.7%)	4 (30.8%)	2 (25.0%)		
Temporal Dynamics							
Time to peak zMAX (days), median [IQR]	-	2.20 (2.00–5.00)	3.00 (2.00–7.00)	15.00 (3.00–35.50)	51.00 (29.75–283.00)	<0.001	<0.001
Recovery time‡ (days), median [IQR]	-	16.00 (7.00–32.00)	69.00 (21.00–136.00)	270.00 (145.00–748.00)	946.00 (586.00–1,051.00)	<0.001	<0.001

Values in the Z-score dynamics section represent the maximum Z-score at each individual visit (visit-specific maximum), not the cumulative maximum. Cumulative maximum Z-scores were used in the Cox analyses.

**Figure 2:**
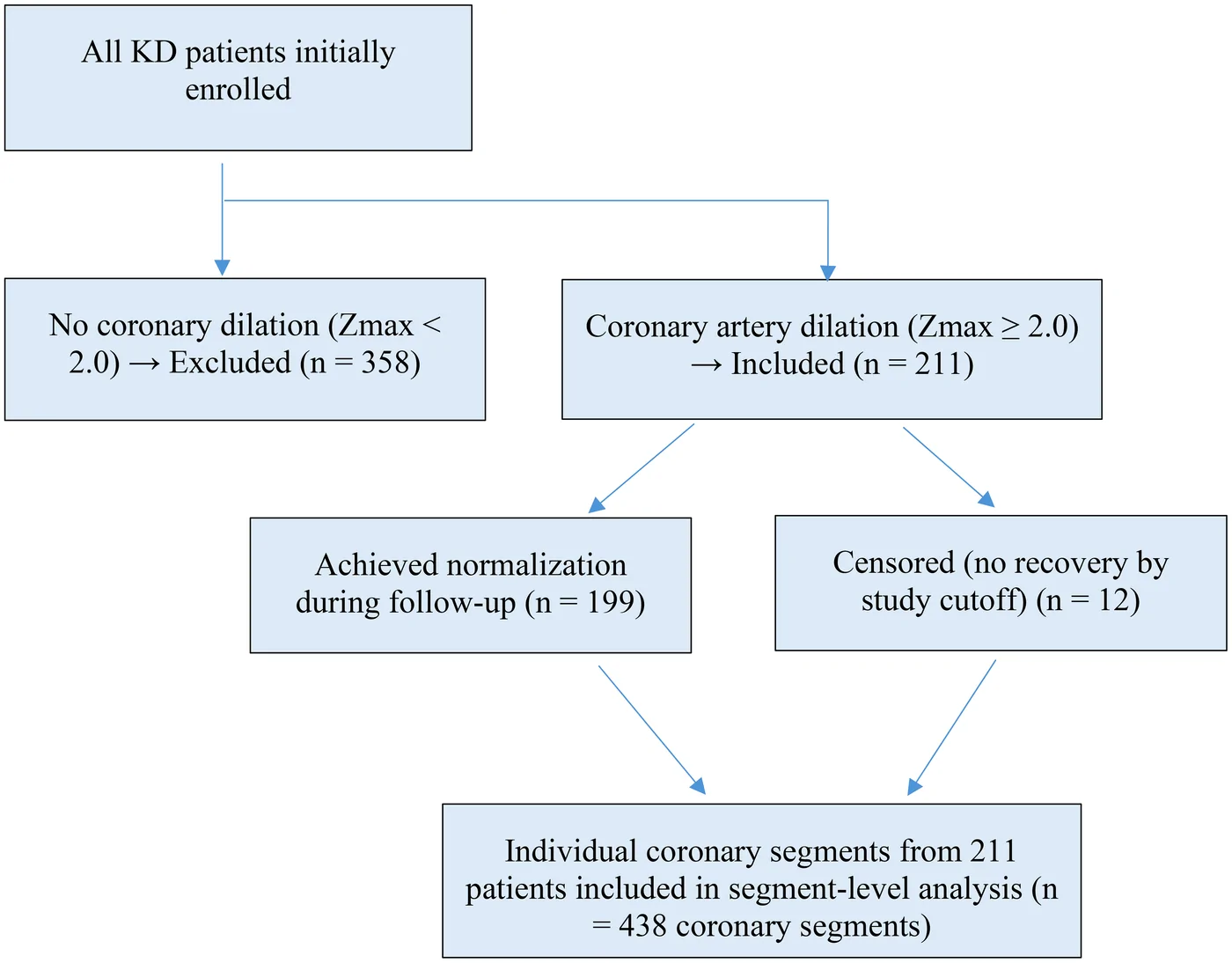
Study flow diagram.

Follow-up duration and censoring: The median follow-up duration for the entire cohort of 569 patients was 400 days (IQR 225.5–750.5 days). For the 211 patients with coronary artery dilation, the median follow-up duration was 558 days (IQR 359.0–852.0 days). Among the 211 patients with dilation, 199 patients (94.3%) achieved normalization of coronary artery diameter by the end of follow-up, including two patients who experienced transient normalization followed by recurrent dilation but ultimately recovered. The remaining 12 patients (5.7%) were right-censored, with a median follow-up duration of 980 days (IQR 763.75–1,051.75 days), indicating that these patients had been followed for nearly three years on average without achieving normalization. These patients remain under active surveillance and continue to undergo scheduled follow-up evaluations beyond the study cutoff date. No patients were lost to follow-up in this cohort.

#### Demographic and seasonal onset characteristics

Significant differences in median age were observed among patient groups across different severity categories. There was a significant negative correlation between disease severity and age at onset (B = −3.210, *P*-trend = 0.007), indicating that for each one-level increase in severity grade, the median patient age decreased by approximately 3.2 months. No significant differences were found in the season of onset or sex distribution among the different severity groups.

#### Dynamic evolution trajectory of coronary artery Z-scores

In this section, the reported Z-scores represent the maximum Z-score at each individual visit (visit-specific maximum) and are used to describe the dynamic evolution of lesions. Among the 211 patients with coronary artery dilation: The Z values of each group of the children showed different evolution patterns. Dilation group: Z-scores showed a continuous downward trend after diagnosis. The median Z-score in the dilation group decreased from an initial 2.19–2.03 after 2 weeks, and further to 1.67 at 1 month, indicating a favorable recovery trend. Aneurysm groups (small, medium, giant): Z-scores remained at relatively high levels. Small aneurysm group: Z-scores slowly decreased from 2.82 (initial) to 2.21 (1 month). Medium aneurysm group: Z-scores remained high throughout the acute phase, even showing a slight increase (from 5.20 to 5.67 at 1 month), followed by a slow decline during the subacute phase. Giant aneurysm group: Z-scores showed a persistently progressive and malignant trend. The median Z-score increased significantly from an initial 9.93 (3.63–12.15) to 12.89 at 1 month and 11.64 at 3 months, indicating that lesions in this group remained extremely active even after the acute phase.

#### Coronary artery involvement pattern

LCX involvement was more frequently observed in severe disease (2.8% in the dilation group vs. 62.5% in the giant aneurysm group). RCAmed involvement was present in all patients in the giant aneurysm group.

### Anatomical distribution pattern of coronary artery lesions and inter-segment correlation

#### Differences in risk of involvement among different coronary artery segments

The overall test of the GEE model indicated significant differences in the risk of involvement among different coronary artery segments. Using RCAmed (the mid-segment of the right coronary artery) as the reference, the risk of involvement of LAD was significantly higher than that of the reference, being 1.87 times the risk of the reference. The risk of involvement of LCX was significantly lower than that of the reference group, with an 81% reduction in risk. The risk of involvement of LMCA and RCA showed no statistically significant difference compared to the reference group (Table 2).

**Table 2:** Parameter estimates.

Parameter	B	Std. Error	95% Wald Confidence Interval		Hypothesis Test			Exp(B)	95% Wald Confidence Interval for Exp(B)	
			Lower	Upper	Wald Chi-Square	df	Sig.		Lower	Upper
(Intercept)	−1.633	0.1134	−1.855	−1.411	207.421	1	<0.001	0.195	0.156	0.244
LMCA	−0.079	0.1446	−0.363	0.204	0.300	1	0.584	0.924	0.696	1.227
RCA	−0.093	0.1018	−0.292	0.107	0.831	1	0.362	0.911	0.746	1.113
LAD	0.624	0.1151	0.398	0.849	29.337	1	<0.001	1.866	1.489	2.338
LCX	−1.680	0.2245	−2.119	−1.240	55.993	1	<0.001	0.186	0.120	0.289
RCAmed	0^a^							1		
(Scale)	1									

#### Inter-segment correlation analysis suggests an adjacent-segment correlation pattern

Analysis of the working correlation matrix suggested a statistical pattern of adjacent-segment correlation of lesions along the vessel axis (Table 3). The matrix showed that correlations between segments within the same vascular system were generally higher than those between different vascular systems.

**Table 3:** Working correlation matrix.

Measurement	Measurement				
	LMCA	RCA	LAD	LCX	RCAmed
LMCA	1.000	0.327	0.486	0.319	0.210
RCA	0.327	1.000	0.425	0.214	0.614
LAD	0.486	0.425	1.000	0.275	0.402
LCX	0.319	0.214	0.275	1.000	0.279
RCAmed	0.210	0.614	0.402	0.279	1.000

Within the left anterior descending artery (LAD) system: The involvement of the proximal (LMCA) and distal (LAD) segments showed a strong positive correlation (correlation coefficient *ρ* = 0.486), suggesting adjacent-segment correlation of the lesion within the LAD vessel. Within the right coronary artery (RCA) system: The involvement of the proximal (RCA) and distal (RCAmed) segments showed the strongest positive correlation (correlation coefficient *ρ* = 0.614). This is consistent with adjacent-segment correlation within the main RCA. Comparison across vascular systems: Correlations between different vascular systems were generally weaker than within-system correlations, further supporting a pattern of adjacent-segment correlation.

### Analysis of factors influencing coronary artery recovery time: patient-level cox proportional hazards model

#### Relative model fit of cumulative maximum Z-scores at different time windows

The cumulative maximum Z-scores at diagnosis (Zmax-T1), within 2 weeks after diagnosis (Zmax-T2), within 1 month after diagnosis (Zmax-T3), within 3 months after diagnosis (Zmax-T4), and the global maximum Z-score (Zmax-Global) during the entire follow-up were entered as continuous variables in separate univariate Cox models. All Zmax variables were highly significant predictors of recovery time (Table 4). The hazard ratios were numerically similar across all time windows, ranging from 0.50 to 0.62.

**Table 4:** Overall cox survival analysis model for patients.

Characteristic	HR	95% CI	*p*-value
Zmax-T1	0.62	0.53,0.72	<0.001
Zmax-T2	0.50	0.42,0.61	<0.001
Zmax-T3	0.52	0.43,0.62	<0.001
Zmax-T4	0.52	0.43,0.63	<0.001
Zmax	0.52	0.43,0.62	<0.001
Age	1.00	1.00,1.01	0.764
Female vs. male	1.37	1.01,1.85	0.041
Coronary segments involved			
1 segment	Reference	-	-
2 segments	0.56	0.39,0.79	0.001
3 segments	0.34	0.23,0.50	<0.001
4 segments	0.22	0.13,0.38	<0.001
5 segments	0.19	0.08,0.44	<0.001
Overall *P* for segment count	-	-	<0.001
Season of Onset			
Spring (Mar-May)	Reference	-	-
Summer (Jun-Aug)	1.17	0.80,1.72	0.415
Autumn (Sep-Nov)	1.19	0.82,1.72	0.370
Winter (Dec-Feb)	0.84	0.53,1.32	0.440
Overall *P* for season	-	-	0.373

Zmax-T1 through T4 and Zmax-Global were analyzed in separate univariate Cox models. Age, sex, segment count, and season were analyzed in separate models as indicated. For the multivariable model containing both Zmax-T2 and segment 1 count, see Results text.

For segment count, the reference category was single- segment involvement (1 segment).

For sex, male was used as the reference category.

Zmax-Global is reported for descriptive purposes only; it incorporates information from the entire follow-up period and was not used as an early prognostic indicator.

The AIC values were: Zmax-T1 1,729.997, Zmax-T2 1,687.148, Zmax-T3 1,683.744, Zmax-T4 1,681.646, and Zmax-Global 1,680.530. Zmax-T2 showed a substantially better fit than Zmax-T1 (*Δ*AIC = 42.8, very strong evidence). According to our predefined criteria, the AIC differences between T2 and T3/T4 (*Δ*AIC = 3.4 and 5.5, respectively) indicate positive evidence, while the difference between T2 and Zmax-Global (*Δ*AIC = 6.6) indicates strong evidence. However, Zmax-Global incorporates information from the entire follow-up period and therefore cannot serve as an early prognostic indicator. We therefore focus on the early time windows (T1–T4) for early risk stratification. Among these, Zmax-T2 provides clinically useful prognostic information at a substantially earlier time point, with only small differences in relative model fit compared to T3 and T4. These findings indicate that Zmax-T2 provides clinically useful early prognostic information, with hazard ratios numerically similar to those obtained at later time points, but with the critical advantage of early availability (within 2 weeks after diagnosis). Accordingly, Zmax-T2 may represent a practical early time window for risk stratification, not because it is statistically superior to later windows, but because it offers useful prognostic information at a substantially earlier time point. These findings suggest that the second week after diagnosis is a clinically useful early time point for risk stratification.

After excluding inter- segment influences, single-segment analysis (data from 438 coronary segments) also showed that the peak coronary Z-score at each stage was significantly correlated with recovery time (Table 5).

**Table 5:** The results of cox regression analysis on the impact of the cumulative maximum Z value in different time windows on recovery time (single-segment model).

Characteristic	HR	95% CI	*p*-value
Zmax-T1	0.51	0.45, 0.59	<0.001
Zmax-T2	0.44	0.38, 0.51	<0.001
Zmax-T3	0.44	0.37, 0.52	<0.001
Zmax-T4	0.43	0.36, 0.52	<0.001
Zmax	0.42	0.37, 0.49	<0.001

CI, Confidence Interval; HR, Hazard Ratio.

#### Impact of the number of involved coronary segments, demographic factors, and season

Compared with single- segment involvement, recovery was significantly slower beginning in the two-segment category. The average hazard of coronary normalization was reduced by 44.4% in patients with two involved coronary segments, and further decreased with an increasing number of involved coronary segments; patients with 5 involved coronary segments had a hazard of coronary normalization only 19.2% that of patients with single-segment disease. Female sex was associated with faster recovery compared with male sex (HR = 1.369, *P* = 0.041), whereas age at onset and season had no significant effect on recovery time (Table 4).

To formally evaluate whether the prognostic effect of coronary segment count remained associated with recovery after adjustment for Zmax severity, we constructed a multivariable Cox model simultaneously entering Zmax-T2 and number of involved coronary segments as covariates. Both variables remained significant in the multivariable model: Zmax-T2 (HR = 0.570, 95% CI: 0.473–0.686, *P* < 0.001) and number of involved coronary segments (HR = 0.770, 95% CI: 0.661–0.896, *P* < 0.001), indicating that segment count remained associated with recovery after adjustment for Zmax-T2. Of note, because the proportional hazards assumption was not fully met for segment count (Schoenfeld *P* = 0.002), this HR should be interpreted as the average effect over the entire follow-up period rather than a constant effect over time; residual confounding cannot be excluded.

Multicollinearity was assessed using the variance inflation factor (VIF), which was 1.170 for both predictors (tolerance = 0.854), well below the conventional threshold of 5, indicating no significant collinearity. The proportional hazards (PH) assumption was evaluated using Schoenfeld residuals. For the primary predictors Zmax-T2 and coronary segment count, the Schoenfeld test suggested evidence of time-varying effects (Zmax-T2: rho = 0.146, *P* = 0.039; segment count: rho = 0.215, *P* = 0.002), indicating that the PH assumption was not fully satisfied. To address this violation, we performed a supplementary time-varying coefficient analysis for Zmax-T2 by incorporating a log(time) interaction term using the time-dependent covariate Cox regression procedure in SPSS (Cox w/Time-Dep Cov). This analysis suggested that the log hazard ratio for Zmax-T2 followed the function: *β*(t) = −2.243 + 0.330 × ln(days). Correspondingly, the hazard ratio associated with Zmax-T2 was 0.106 at day 1, 0.326 at 30 days, 0.744 at 365 days, and approached 1.0 at approximately 900 days (about 2.5 years), suggesting that the prognostic effect of Zmax-T2 is strongest in the acute/subacute phase and gradually attenuates over time. The standard Cox model (HR = 0.570) is therefore best interpreted as the average effect over the entire follow-up period. The standard Cox model (providing the overall average hazard ratio for both predictors) and the time-varying coefficient model (providing the dynamic trajectory for Zmax-T2) were analyzed in parallel, with the former serving as a global summary and the latter as a refined characterization.

#### Landmark analysis for time-dependent bias

To examine whether the prognostic value of Zmax was influenced by time-dependent bias, we performed landmark analyses at diagnosis, 2 weeks after diagnosis, 1 month after diagnosis, and 3 months after diagnosis. As shown in Table 6, at all four landmark time points, the visit-specific maximum Z-score at each landmark visit remained a significant predictor of subsequent recovery among patients who had not yet achieved normalization at each respective time point. The hazard ratios (HRs) were 0.578 (95% CI: 0.488–0.686, *P* < 0.001) at diagnosis, 0.630 (95% CI: 0.530–0.749, *P* < 0.001) at 2 weeks, 0.641 (95% CI: 0.534–0.769, *P* < 0.001) at 1 month, and 0.758 (95% CI: 0.642–0.895, *P* = 0.001) at 3 months.

**Table 6:** Landmark analysis of the association between the visit-specific maximum Z-score at each landmark visit and subsequent coronary recovery at four prespecified time points.

Landmark time point	Patients at risk, n	Events, *n* (%)	HR (95% CI) for Zmax	*P*-value
Diagnosis (T1)	211	199 (94.3%)	0.578 (0.488–0.686)	<0.001
2 weeks (T2)	159	147 (92.5%)	0.630 (0.530–0.749)	<0.001
1 month (T3)	100	88 (88.0%)	0.641 (0.534–0.769)	<0.001
3 months (T4)	45	34 (75.6%)	0.758 (0.642–0.895)	0.001

At each landmark time point, only patients who had not yet achieved normalization and remained under observation were included. Survival time was recalculated from the landmark time point to the date of subsequent recovery. HRs were derived from separate Cox proportional hazards models for each landmark time point, with the visit-specific maximum Z-score at each landmark visit as the respective predictor.

Notably, the HRs gradually approached unity as the landmark time point advanced (from 0.578 at diagnosis to 0.758 at 3 months), with a corresponding progressive reduction in sample size (from 211 to 45) and an increasing censoring proportion (from 5.7% to 24.4%). This pattern is consistent with the expected natural history of the disease: patients with milder lesions recovered earlier and were excluded from later landmark analyses, leaving a progressively more severe and homogeneous population in whom the association between Zmax and subsequent recovery attenuates over time. Nevertheless, the persistence of statistically significant associations across all landmark time points suggests that the predictive value of Zmax is robust and unlikely to be solely explained by time-dependent bias, although residual confounding by unmeasured time-varying factors cannot be entirely excluded.

#### Inter-observer reproducibility

Inter-observer reproducibility was excellent for all five coronary segments. The ICCs (single measures, 95% CI) were 0.941 (0.891–0.968) for LMCA, 0.965 (0.936–0.982) for LAD, 0.928 (0.867–0.961) for LCX, 0.976 (0.956–0.987) for RCA, and 0.940 (0.890–0.968) for RCAmed (all *P* < 0.001). These findings suggest that measurement variability between observers was minimal, supporting the reliability of our Z-score measurements.

## Discussion

Through a retrospective analysis of 569 children with Kawasaki disease, of whom 211 had coronary involvement, this study systematically investigated the anatomical distribution pattern of coronary lesion and the predictive value of dynamic Z-scores for recovery time. The strength of this study lies in the systematic continuous echocardiographic follow-up of these five standard coronary segments, with strict assessment using body surface area-standardized Z-scores, providing a valuable single-center echocardiographic dataset based on a Chinese population.

### Anatomical distribution pattern of coronary artery lesions and its significance

Using a Generalized Estimating Equation (GEE) model, this study suggested a statistical pattern of adjacent-segment correlation in coronary artery lesions, with the strongest correlations observed within the LAD and RCA systems. This observation is consistent with the hypothesis that vascular injury may follow an axial distribution, as previously suggested by pathological studies (18). However, the GEE working correlation matrix itself does not establish a causal or mechanistic propagation process; it primarily characterizes within-patient correlation patterns. Previously, Liu et al. (19) indicated that multi-segment involvement is associated with poor prognosis but lacked elaboration on its underlying pattern. This finding suggests that such multi-segment involvement may more often reflect correlated involvement of adjacent segments within a single vascular system, although the GEE analysis does not establish lesion propagation or distinguish among possible biological mechanisms. This pattern suggests that during echocardiographic evaluation, if proximal involvement of a coronary segment is found, careful assessment of its distal segments may be clinically useful.

### Prognostic value of dynamic Z-scores and a clinically useful early assessment time window

We found that the AIC values for Zmax-T2, Zmax-T3, Zmax-T4, and Zmax-Global were similar (range: 1,680.530–1,687.148). However, Zmax-Global includes information from the entire follow-up period and is not suitable as an early predictor. Among the early time windows, Zmax-T2 showed a marked improvement over the initial-evaluation Zmax-T1 (*Δ*AIC = 42.8), whereas the differences between T2 and T3/T4 were small (*Δ*AIC = 3.4 and 5.5), supporting its practical value for early assessment. These findings indicate that the assessment around the second week after diagnosis provides clinically useful prognostic information, with hazard ratios numerically similar to those from later measurements (1 month or 3 months), but at a substantially earlier time point. However, this numerical similarity does not establish equivalence in relative model fit. This suggests that although the inflammatory peak may vary across individuals and often extends beyond the second week in severe cases, the assessment around the second week may capture an early burden of vascular injury that already provides meaningful prognostic information, suggesting that it may represent a clinically practical time window for early risk stratification.

Previous studies have established that Z-scores measured at diagnosis are strong predictors of coronary outcomes (11, 12, 15). However, these studies primarily focused on a single baseline measurement and did not systematically evaluate whether later time points offered additional prognostic information. Our finding that Zmax-T2 provides clinically useful prognostic information, with hazard ratios numerically similar to those from later time points, suggests that key prognostic signals may be discernible by the second week—well before the conventional 1-month or 3-month follow-up assessments. This temporal precision may have clinical implications, suggesting that it could enable early risk stratification without waiting for later confirmatory imaging.

The prognostic value of early Z-score assessment has also been reported in other populations. Suzuki et al. (20) reported that a pre-treatment Z-score ≥1.6 was the strongest predictor of post-treatment coronary artery abnormalities in a Japanese cohort (OR 2.25–9.98). La Vecchia et al. (21) found that a baseline Z-score ≥2 was significantly associated with CAA at 1-month and 2-month follow-ups in a European cohort. Xu et al. (22) further showed that baseline Zmax independently predicted CAL progression (OR 1.51), and additionally found the number of involved coronary segments to be associated with outcomes (OR 2.32)—a finding that parallels our observation regarding multi-segment involvement. While these studies consistently support the predictive utility of early Z-scores, none directly compared the relative model fit across multiple early time windows to identify a time point at which prognostic information may stabilize. Our AIC-based comparison suggests that Zmax-T2 provides clinically useful early prognostic information, with hazard ratios numerically similar to those obtained from later measurements, suggesting that the second week after diagnosis may represent a practical early time window for risk stratification.

Notably, the Schoenfeld residual analysis suggested a time-varying effect of Zmax-T2, which we formally modeled using a log(time) interaction term. This modeling approach suggested that the prognostic impact of early coronary Z-score is not uniform over time. Instead, its effect is strongest in the acute/subacute phase and diminishes as time elapses, consistent with the dynamic nature of vascular remodeling. The standard Cox model provided an overall hazard ratio of approximately 0.50 for Zmax-T2 (Table 4), representing the average effect over the entire follow-up period. However, this average estimate masks the underlying temporal variation: the instantaneous effect was much stronger early after diagnosis (HR ≈ 0.106 at day 1) and gradually weakened to near-null by approximately 2.5 years. This finding suggests the importance of early risk stratification and suggests that the timing of Z-score measurement may be important for predicting recovery. The early (2-week) window may reflect the early burden of vascular injury, which appears to be associated with the subsequent remodeling trajectory. The fact that the effect diminishes over time does not necessarily undermine the clinical value of early Z-score assessment; rather, it is consistent with the hypothesis that the acute phase may represent a potential time window for early risk stratification. However, this interpretation remains speculative and requires confirmation through prospective studies with systematic inflammatory biomarker measurements and longer-term outcome validation.

### Extent of lesions, demographic factors, and insights from the Chinese population data

The observed prevalence of coronary artery dilation (37.1%) in our cohort warrants specific discussion. First, the definition of CAL in this study followed the American Heart Association criteria using a Z-score cutoff of ≥2.0, which is considerably more sensitive than the absolute diameter criteria of the Japanese Ministry of Health. Notably, a recent large-scale study from the UCSD Kawasaki Disease Research Center involving 1,016 KD patients reported a coronary artery abnormality rate of 37.8% using the same AHA Z ≥ 2.0 criteria—almost identical to our 37.1% (23). This consistency suggests that our CAL rate is not anomalously high, but rather reflects the expected prevalence under the more sensitive Z-score-based criteria. Second, as a tertiary referral center, our institution may have disproportionately enrolled patients with more severe disease or those referred for complicated management, potentially inflating the observed CAL rate compared with population-based cohorts.

This study suggested that the number of involved coronary segments remained associated with recovery after adjustment for Zmax-T2, although residual confounding cannot be excluded. Importantly, this segment count variable incorporates information obtained after the early phase, and it is therefore described as a marker of overall lesion extent rather than an early prognostic indicator. The early prognostic indicator in our study is Zmax-T2. A greater number of involved coronary segments was associated with a significantly reduced hazard of coronary normalization, exhibiting a graded association when interpreted as the average effect over the entire follow-up period (as noted above, the proportional hazards assumption was not fully met for coronary segment count, and the single HR represents the average effect rather than a constant effect over time). Compared with single-segment involvement, recovery was significantly slower beginning in the two- segment category. Patients with 5 involved coronary segments had a hazard of coronary normalization only 19.2% that of patients with single-segment disease. This is consistent with the findings of another study by Liu et al. (19), highlighting the necessity of comprehensively assessing both “lesion severity” and “lesion extent” in risk stratification.

Large-scale epidemiological data indicate that sex, season, and age are known risk factors for coronary artery complications in Kawasaki disease (24). In our cohort, sex was significantly correlated with recovery time, whereas season and age were not. Specifically, female sex was associated with faster recovery (HR = 1.369, *P* = 0.041), suggesting that male sex is a risk factor for delayed recovery.

This sex difference has been observed in other populations and is increasingly supported by biological evidence. Watelle et al. (25), in a Canadian multicenter study, reported that males have more than twice the risk of developing medium to large aneurysms compared with females (adjusted risk difference 4.6%), and since most subjects were prepubertal, they suggested that sex hormones alone do not explain the disparity. More importantly, Kim et al. (26) identified six male-specific CAA susceptibility loci (e.g., NOS3, PDE1C) through sex-stratified GWAS, providing a genetic basis for the observed sex difference. Zhang et al. (27) further identified sex, rash symptoms, delayed IVIG treatment, CAL type and degree, and pre- and post-IVIG platelet counts as significant factors associated with overall CAL duration. While these studies consistently point to male sex as a risk factor for more severe coronary involvement, our study offers a recovery-time perspective: the male predisposition to worse coronary outcomes may translate into a measurable delay in recovery time—a clinically meaningful endpoint that adds to the existing evidence on aneurysm formation and CAL duration.

It is worth pointing out that this study, based on data from Chinese children, provides further evidence on the natural history of coronary artery lesions in Kawasaki disease in East Asian populations. Previously, de Zorzi et al. (8) and Liu et al. (19) have indicated that using body size-based Z-score criteria is more sensitive in identifying coronary artery abnormalities and avoids underestimation compared to absolute diameter criteria.

### Limitations

This study has several limitations. First, as a single-center retrospective study conducted at a tertiary referral center, selection bias may exist, and the high proportion of patients with coronary artery lesions may not be representative of the general KD population. Consequently, the observed prognostic associations—particularly the predictive value of Zmax-T2 and the association observed for coronary segment count—may not be directly generalizable to community-based or lower-risk Kawasaki disease populations. The generalizability of our findings to primary care or community-based settings should therefore be interpreted with caution. Second, although the currently recommended Z-score system was used, differences between different formulas within this cohort were not directly compared; future research could explore this. Third, the primary outcome was defined as morphological recovery on ultrasound, lacking longer-term endpoints such as cardiac function, myocardial perfusion, or cardiovascular events in adulthood. Fourth, although we performed an inter-observer reproducibility analysis on a randomly selected subset of 40 patients using archived images, which demonstrated excellent agreement for all five coronary segments (ICCs ranging from 0.928 to 0.976, all *P* < 0.001), we acknowledge that formal intra-observer reproducibility was not assessed due to the retrospective nature of this study. Nonetheless, the high inter-observer reproducibility supports the reliability of our Z-score measurements and substantially mitigates concerns regarding measurement variability.

Fifth, the follow-up intervals were individualized according to lesion severity, with more severe lesions receiving more frequent echocardiographic assessments per guideline recommendations. This differential surveillance intensity could theoretically introduce surveillance bias. However, several factors mitigate this concern: our follow-up schedule followed established AHA and JCS guidelines rather than investigator discretion; the recovery times for moderate and giant aneurysms were substantially prolonged (median 270 and 946 days, respectively), whereas intensified early surveillance windows occurred at time points when biological recovery of these severe lesions was impossible; and the 12 censored patients had a median follow-up of 980 days without normalization, supporting the robustness of our censoring classification. Nonetheless, we acknowledge this limitation and encourage future prospective studies with protocol-mandated follow-up schedules to further validate our findings.

Sixth, although multiple imputation was used to handle missing data with modest overall missing proportions (maximum 11.4% at T4), we further performed a complete-case sensitivity analysis, which yielded results consistent with the primary analysis (all HRs ranging from 0.52 to 0.62, *P* < 0.001). However, we cannot entirely exclude the possibility of non-random missingness, which may have introduced some degree of bias.

Seventh, the multivariable models did not adjust for key clinical and treatment-related covariates such as time from fever onset to IVIG treatment, IVIG resistance, adjunctive therapies (corticosteroids, infliximab), laboratory parameters, or complete versus incomplete Kawasaki disease, as these data were not systematically captured in this retrospective cohort. The lack of adjustment for these factors may affect the assessment of independent prognostic value. Future prospective studies with comprehensive covariate collection are needed to validate and extend our findings. Additionally, although we performed stratified Kaplan–Meier analyses, the confidence intervals were substantially wide across strata, likely reflecting the multifactorial nature of coronary recovery. This highlights the need for multivariable risk prediction models incorporating both imaging and clinical covariates—a direction we are planning to address in our ongoing research. Therefore, to reflect the limited covariate adjustment and to avoid overinterpretation, we describe our findings as ‘remained associated with recovery after adjustment for Zmax-T2’ rather than ‘independent prognostic factors’.

Eighth, to address potential time-dependent bias when using later-window Zmax variables, we performed landmark analyses at diagnosis, 2 weeks, 1 month, and 3 months, including only patients who remained unrecovered at each time point. These analyses suggested that Zmax remained significantly predictive of subsequent recovery at all landmarks (all *P* ≤ 0.001), suggesting robustness of our findings against time-dependent bias while acknowledging that residual confounding by unmeasured time-varying factors cannot be entirely excluded. The sample size at the 3-month landmark was limited (*n* = 45). Future prospective studies with larger samples are warranted to further validate these findings.

Ninth, the time-varying coefficient model was implemented using a prespecified log(time) interaction term, which assumes a specific parametric form for the temporal variation of the effect. While this approach is widely used and provided a reasonable fit to our data, other functional forms (e.g., splines or step functions) might capture additional nuances. However, the consistency between the time-varying coefficient findings and the landmark analyses suggests the robustness of our conclusions regarding the dynamic prognostic value of Zmax-T2. Additionally, we acknowledge that the Schoenfeld residual analysis also suggested a time-varying effect for segment count (rho = 0.215, *P* = 0.002). However, segment count was entered as a continuous variable in the multivariable model to estimate the effect per one-segment increase, but it is inherently a count variable (1–5 coronary segments), and modeling a time-varying coefficient for such a variable poses substantial challenges in both estimation and interpretation. The multivariable Cox model, which provides an overall average effect, remains a widely accepted approach for evaluating whether lesion extent remained associated with recovery after adjustment for Zmax severity, while we acknowledge that this estimate does not characterize how the association changes over time and that residual confounding cannot be excluded. Future studies with larger samples are needed to further characterize the temporal dynamics of lesion extent.

## Conclusion

Based on echocardiographic data from a Chinese population, this study suggested a statistical pattern of adjacent-segment correlation of coronary artery lesions along the vessel axis in Kawasaki disease. Lesion involvement showed strong axial correlations within the LAD system (*ρ*=0.486) and RCA system (*ρ*=0.614), while correlations between different vascular systems were weak. The study also suggested the value of Z-score assessment in the acute phase, particularly within two weeks after diagnosis, for predicting coronary artery recovery time. Formal model comparison using AIC suggested that Zmax-T2 provides clinically useful early prognostic information, with hazard ratios numerically similar to those obtained at 1 month or 3 months (HRs 0.52 for both), while offering the advantage of substantially earlier availability. Compared with the diagnosis-time Zmax-T1, Zmax-T2 showed a markedly superior model fit (*Δ*AIC = 42.8), suggesting its value as a practical early assessment window. Using Zmax-T2 as the time window, each 1-unit increase in the coronary artery Z-score was associated with an approximately 49.6% reduction in the hazard of coronary normalization (HR ≈ 0.504). Furthermore, time-varying coefficient analysis suggested that the prognostic effect of Zmax-T2 is dynamic: it is strongest in the acute/subacute phase and gradually attenuates over time, approaching null at approximately 2.5 years. This dynamic pattern is consistent with the hypothesis that early risk stratification is clinically relevant and does not necessarily undermine the clinical utility of the 2-week assessment window. The study further suggested that the extent of lesions (number of involved coronary segments) remained associated with recovery after adjustment for Zmax-T2, although residual confounding cannot be excluded and this association should be interpreted as an average effect over the entire follow-up period (as the proportional hazards assumption was not fully met for coronary segment count; this estimate does not characterize how the association changes over time). Compared with single-segment involvement, recovery was significantly slower beginning in the two-segment category. Patients with 5 involved coronary segments had a hazard of coronary normalization only 19.2% that of patients with single-segment disease. These findings provide evidence-based medical support for precise risk stratification, individualized follow-up strategy formulation, and future targeted interventional studies for coronary artery lesions in Kawasaki disease.

## Data Availability

The raw data supporting the conclusions of this article will be made available by the authors, without undue reservation.
